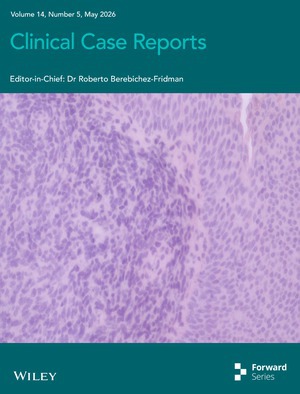# Cover Image

**DOI:** 10.1002/ccr3.72775

**Published:** 2026-05-19

**Authors:** George Evele, Francine Kouya, George Ngock, Richard Bardin

## Abstract

The cover image is based on the article *Clinicopathological Characteristics and Outcomes of Genitourinary Rhabdomyosarcoma in Two Girls* by George Evele et al., https://doi.org/10.1002/ccr3.72691.